# The overexpression of genes of thiol metabolism contribute to drug resistance in clinical isolates of visceral leishmaniasis (kala azar) in India

**DOI:** 10.1186/s13071-014-0596-1

**Published:** 2014-12-17

**Authors:** Neeloo Singh, Mitali Chatterjee, Shyam Sundar

**Affiliations:** Central Drug Research Institute, Jankipuram Extension, Lucknow, 226031 India; Department of Pharmacology, Institute of Post Graduate Medical Education and Research, Kolkata, India; Banaras Hindu University, Varanasi, India

**Keywords:** *Leishmania donovani*, Drug resistance, Drug efflux, Thiols, Clinical isolates

## Abstract

**Background:**

Visceral leishmaniasis (VL), also called Kala Azar (KA) or black fever in India, claims around 20,000 lives every year. Chemotherapy remains one of the most important tools in the control of VL. Current chemotherapy for Kala Azar in India relies on a rather limited arsenal of drugs including sodium antimony gluconate and amphotericin B in addition to the very expensive drug miltefosine. Pentavalent antimonials have been used for more than half a century in the therapy of leishmaniasis as it is relatively safe and inexpensive, however, the spread of resistance to this drug is forcing clinicians in India to abandon this treatment. Consequently, improvement of antimonial chemotherapy has become a major challenging area of study by leishmaniacs worldwide. The alarming emergence of resistance to the commonly used antleishmanial drug, sodium antimony gluconate, in India, has led us to elucidate the resistance mechanism(s) in clinical isolates. Studies on laboratory mutants have shown that resistance to antimonials is highly dependent on thiol levels. The parasite evades cytotoxic effects of antimonial therapy by enhanced efflux of drug upon conjugation with thiols, through overexpressed membrane proteins belonging to the superfamily of ABC transporters.

**Methods:**

We have carried out functional studies to determine the activity of the efflux pumps in antimonial resistant clinical isolates collected from disease endemic areas in India and also carried out molecular characterization of thiol levels in these parasites.

**Results:**

Overexpression of the gene coding for γ glutamylcysteine synthetase was observed in these resistant clinical isolates thereby establishing that thiols represent the key determinants of antimonial resistance. The SbIII/thiol conjugates can be sequestered by ABC transporter multidrug resistance protein A (MRPA) into intracellular organelles or can be directly pumped out by an uncharacterized transporter.

**Conclusions:**

Our studies investigating antimonial resistance in different *L. donovani* clinical isolates suggest that over functioning of MRP plays a role in generation of antimony resistance phenotype in some *L. donovani* clinical isolates.

## Background

Visceral leishmaniasis (VL, kala-azar) is prevalent in 62 countries with an estimated annual incidence of 500,000 (www.dndi.org). In Eastern India, the State of Bihar and adjoining areas of West Bengal, Jharkhand and Uttar Pradesh account for about half the world’s burden of VL. A recent report by the Press Trust of India (PTI) states ‘Kala Azar stalks Bihar’ and reports that in Patna, the capital city of Bihar, kala azar has affected an estimated 25,000 people and claimed 93 lives. Thirty-one of the state’s 38 districts are in its grip. Although the foci of VL in India is Bihar, West Bengal, Uttar Pradesh and Jharkhand, cases have also been reported from Gujarat (west India) [1], Tamil Nadu and Kerala (south India) [2], and sub- Himalayan parts of north India including Uttar Pradesh, Himachal Pradesh and Jammu and Kashmir [3]. Globally, including India, the treatment of VL has centered around pentavalent antimony compounds (Sb^V^) for more than seven decades, however, about 65 per cent of previously untreated patients are unresponsive rendering the drug useless for routine use [4]*.* Multiple resistance mechanisms have been described in resistant *Leishmania* species developed *in vitro*. The drug SbV can be taken up by the parasite via an unidentified transporter [5,6]. Sb(V), a prodrug, have to be converted to Sb(III) in order to be active. Parasite specific thiol dependent reductase 1 (TDR1) and ACR2 enzymes were characterized in *Leishmania* and was shown to reduce Sb(V) to Sb(III) [7,8]. Alternatively, there is evidence that a number of thiols, including parasite-specific thiols such as trypanothione as well as macrophage-specific thiols such as glycylcysteine, can reduce Sb(V) to Sb(III) non-enzymatically [9]. The resulting SbIII can form conjugates with thiols and inhibit trypanothione reductase (TR) together leading to increase of redox potential [10]. The SbIII/thiol conjugates can be sequestered by ABC transporter multidrug resistance protein A (MRPA) [11] into intracellular organelles or can be directly pumped out by an uncharacterised transporter [12]. Studies investigating antimonial resistance in different *L. donovani* clinical isolates from our lab and others have suggested a very heterogeneous situation. The upregulation of antioxidant pathways in SSG-resistant parasites was most frequently reported [13,14]. Increased expression of enzyme tryparedoxin peroxidase may play an important role in clinical resistance to antimony. Elevated levels of tryparedoxin peroxidase in antimony-unresponsive *L. donovani* field isolates has been reported in *Leishmania braziliensis* and *Leishmania infantum* [15,16]. A comprehensive characterisation of the parasite pathways implicated in SSG/SbV/SbIII metabolism using a collection of parasite strains isolated from Nepalese VL patients established that molecular changes associated with antimonial-resistance in natural *Leishmania* populations depend on the genetic background of the *Leishmania* population, which has resulted in a divergent set of resistance markers in the *Leishmania* populations [17]. Increasing unresponsiveness to Sb^v^ in India, administered as sodium antimony gluconate (SAG), has led to successive advent of new drugs [18] however, all plagued by shortcomings. Affordable and effective chemotherapy is still beyond reach of the common man in India. Consequently, this study has been undertaken to determine the status of efflux pumps and thiols in antimony unresponsive clinical isolates of *L. donovani* collected from the endemic region of Bihar, India. An understanding of resistance mechanism(s) operating in clinical isolates might lead us to bring back this molecule alone or in combination therapy.

## Methods

### Collection of *Leishmania donovani* clinical isolates

In our study patients were selected from the disease non-endemic region of Uttar Pradesh (U.P.) as well as from highly endemic regions viz. Kala Azar Medical Research Centre of the Institute of Medical Sciences, Banaras Hindu University, Varanasi and also from its affiliated hospital situated at Muzaffarpur, Bihar. The Ethics Committee of Kala Azar Medical Research Centre of Banaras Hindu University in Varanasi, India, reviewed and approved the protocol and written informed consent was obtained from the subjects participating in the study. The diagnostic criteria for VL were the presence of LD bodies (Leishman Donovan) in splenic aspirations performed and graded as per standard criteria [19]*.* After diagnosis, the patients were administered a course of sodium antimony gluconate (SAG) [Albert David, Calcutta, India] 20 mg/Kg body weight once daily for 30 days. Response to treatment was evaluated by a repeat splenic aspiration on day 30 of treatment. Patients were designated responsive based on the absence of fever, clinical improvement with reduction in spleen size and absence of parasites in the splenic aspirate while patients who showed presence of parasites in splenic aspirates were labelled as antimonial unresponsive. These patients were subsequently treated successfully with amphotericin B.

### Culture conditions

The splenic aspirates of the responsive and unresponsive patients with VL were inoculated into NNN medium and passaged every seventh day into a tube containing fresh NNN medium. Positive cultures were then progressively adapted to M199 medium supplemented with 15% fetal calf serum for mass culture. The isolates used in this study are listed in Tables 1and 2.

**Table 1:** **Profile of clinical isolates used in the study**

**S. No**	**Isolate**	**Year of collection**	**Place of collection**	**Drug response to SAG**
1	R-5	09.02.1998	Muzaffarpur, Bihar	Unresponsive
2	39	28.05.2000	Muzaffarpur, Bihar	Unresponsive
3	41	28.05.2001	Muzaffarpur, Bihar	Unresponsive
4	2001	01.02.2000	Balia, U.P.	Responsive

**Table 2:** **Profile of clinical isolates used in the study**

**S. No**	**Isolate**	**Year of collection**	**Place of collection**	**Drug response to SAG**
1	93	16.12.2003	Muzaffarpur, Bihar	Unresponsive
2	77	05.11.2003	Muzaffarpur, Bihar	Unresponsive
3	87	06.12.2003	Balia, U.P.	Responsive
4	4	29.11.2004	Balia, U.P.	Responsive
5	138	04.02.2005	Deworia, U.P.	Responsive

### Assay for drug sensitivity

Assay for *in vitro* drug sensitivity to SAG was done as described [20,21]*.* We used amastigotes rather than promastigotes. The promastigote stage in the lifecycle is not susceptible to two major drugs used clinically, these being pentavalent antimonial drugs, sodium stibogluconate and meglumine antimoniate. The virulence and level of susceptibility or resistance of these isolates was also confirmed *in vivo*, by infection in golden hamsters as described [22]*.*

### Nucleic acid isolation and blotting

Nuclear DNA was isolated by established procedure using proteinase K digestion [23]*.* Total RNA was isolated using TRIZOL (Gibco BRL) [24]*.* Probe for γ-glutamylcysteine synthetase gene (GCS) was made from PCR amplified 73 bp GCS fragment using primers as described [25]. Alpha tubulin gene primers obtained as a kind gift from Marc Ouellette, Quebec, Canada were used for amplification and probe preparation. Probes were made by labelling 25 ng of the DNAs with [α-^32^P] dCTP by random priming method (BRIT/BARC, India).

### Flow cytometry

Flow cytometry was employed to ascertain the functionality of ABC transporters in the promastigotes of antimony unresponsive isolates (39, 41, R-5) and responsive isolate (2001). Two substrates, Rhodamine 123 (stock: 3 mM in methanol) was diluted to 100 μM in 0.02 M Phosphate Buffered Saline pH 7.2 (PBS) and used at a final concentration of 1 μM. Calcein AM (1 mM in DMSO) was diluted to a final concentration of 1 μM in medium. To identify the nature of the pump, known modulators of MDR and MRP namely verapamil (Sigma, St. Louis, USA, 4 mM in PBS, final concentration 10 μM) and probenecid (Sigma, St. Louis, USA, 0.4 M in PBS, pH 8.5, final concentration 4 mM) respectively were used.

### Fluorescence labelling of *L. donovani* promastigotes

Log phase promastigotes (2 × 10^6^/ml) were washed twice with medium M199 (Sigma, St Louis, USA) supplemented with 10% FBS, 25 mm HEPES pH 7 · 4 (Medium A) and incubated at 26°C for fluorescence labelling. Both accumulation and retention assays were done as described [26].

Accumulation assays: Promastigotes (2× 10^6^/ml) were initially washed twice in HEPES buffered saline referred to as ‘HBS1’ (21 mM HEPES, 137 mM NaCl, 5 mM KCl, 0.7 mM NaH_2_PO4 and 20 mM glucose, pH 7.4). After addition of Rhodamine 123, fluorescence was measured at 0, 15, 30 and 45 minutes whereas with calcein AM, fluorescence was measured at 0, 15, 30, 45 and 60 minutes. To study influence of energy depletion upon pump activity, accumulation of both fluorochromes was measured. Promastigotes were washed twice in HEPES buffered saline referred to as ‘HBS1’ (21 mM HEPES, 137 mM NaCl, 5 mM KCl, 0.7 mM NaH_2_PO4, 20 mM glucose, pH 7.4). They were transferred to a modified HBS1 where glucose had been specifically excluded and 20 mM sodium azide (NaN) included, referred to as ‘HBS2’ and incubated in HBS2 at 26°C for 30 minutes and transferred to PBS containing the fluorescent substrates in the presence or absence of modulators and readings taken at different time points.

Retention assays: Promastigotes were pre-loaded with calcein by incubating them with Calcein AM for 1 h at 26°C. Cells were centrifuged at 3000 g for 5 minutes at 4°C, then washed twice with ice cold Medium A, immediately re-suspended in same medium with or without probenecid, but notably excluding Calcein AM and placed on ice. Thereafter, first reading was taken and was considered as 0 minute reading. Promastigotes were transferred to 26°C and fluorescence measured at 15, 30, 45, 60 minutes.

### Thiol level detection

To measure intracellular thiol levels, Cell Tracker Green CMFDA (Molecular Probes, stock 10 mM in DMSO; final concentration 10 μM in serum free medium) was used. Promastigotes were incubated with Cell- Tracker both in normal and energy depleted conditions and fluorescence measured after 45 minutes of incubation at 26°C.

### Flow cytometric analysis

Monitoring of dye accumulation and retention was carried out as described earlier [26] on a flow cytometer (FACS Calibur, Becton Dickinson) equipped with an argon-ion laser (15 MW) tuned to 488 nm. Data analysis was carried out with Cell Quest (BD) software. Fluorescence of Rhodamine 123, Calcein AM, and Cell Tracker were collected in the photomultiplier tube designated FL1, which is equipped with a 530/30- nm band pass filter. Filter combination and protocols were used to generate scatter grams and list mode data on forward *vs*. side scatter and counts *vs*. FL1 height. Samples were analyzed at the flow rate of 100-200 cells/second and a typical analysis was based on examination of 10,000 cells. Drug fluorescence was measured on a log scale while cell counts were on a linear scale. Dead cells or cells with compromised membranes were discriminated by adding propidium iodide (PI, 5 μg/ml) in every tube. Fluorescence of PI was taken at photomultiplier tube designated FL2 equipped with a 585/42-nm band pass filter. Each result is a representative of four sets of independent experiments. For experiments using cell tracker, in order to eliminate the individual variance of basal fluorescence of each sample, we have represented the data as the ratio of fluorescence at any time point divided by its 0 minute fluorescence and plotted on the Y-axis.

## Results

### Clinical isolates

During the year 1998-2000, resistance to the widely used antimonial drug sodium antimony gluconate (SAG) had reached alarming heights in India [5]*.* At this time we had cultivated many unresponsive isolates (Table 1). Isolates R-5, 39 and 41 are from patient who did not respond to SAG therapy therefore these are labelled as ‘unresponsive’; isolate 2001 responded to SAG therapy hence ‘responsive’. Since one of the most frequently applied strategies in biological systems against resistance to cytotoxic drugs is the efflux of these compounds from the cell via membrane proteins, in the present study we have used these isolates to ascertain their efflux pump phenotype and to check for amplification and overexpression of gamma glutamylcycteine synthetase (GCS) gene.

### Flow cytometry shows differential expression of ABC transporters between resistant and sensitive isolates

**Figure 1:**
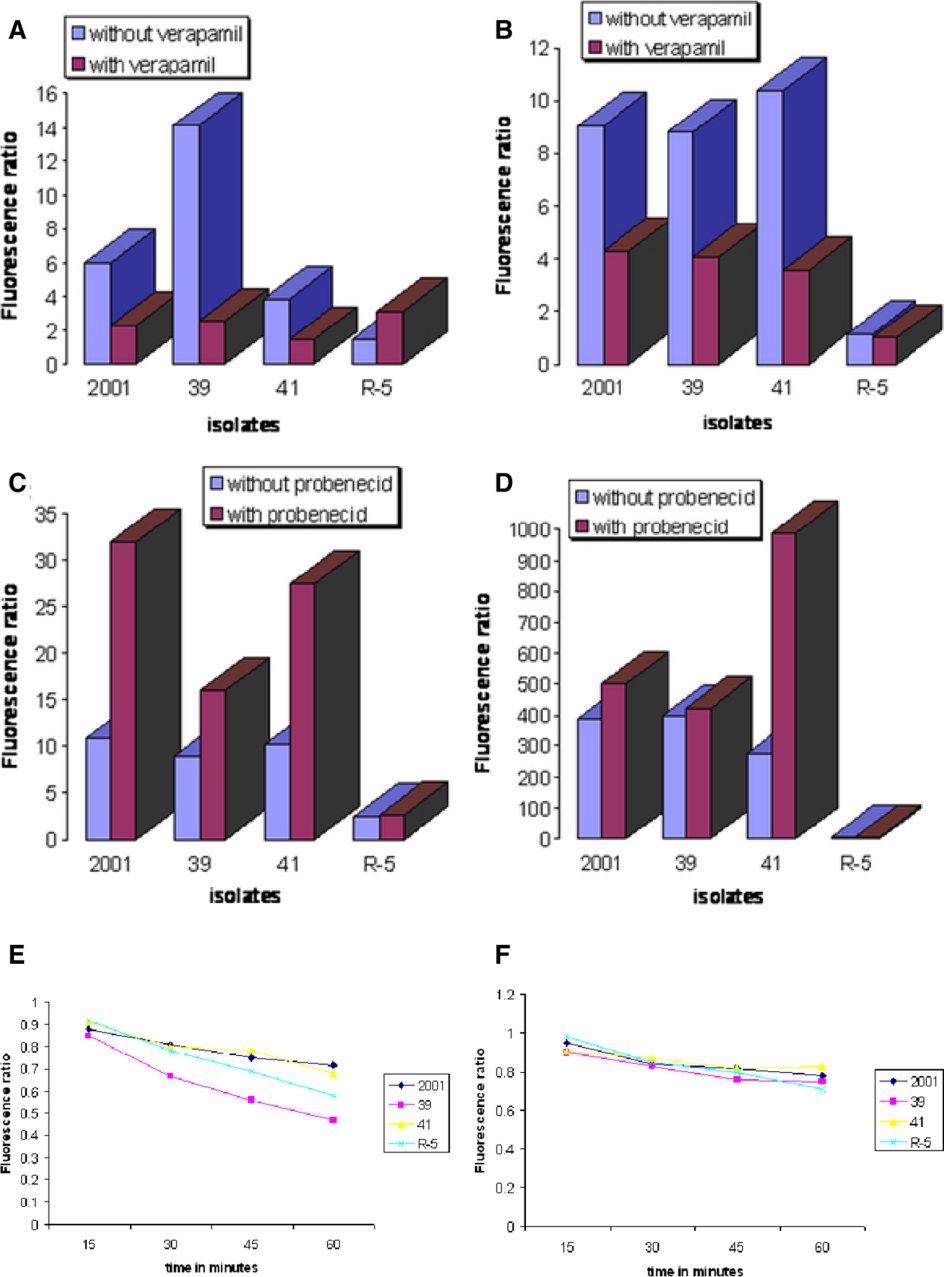
**Comparison of Rhodamine and Calcein accumulation with retention in** ***L. donovani*** **clinical isolates. (A)** Rhodamine accumulation in promastigotes of sensitive (2001) and resistant (39, 41, R-5) isolates under normal condition after 45 minutes of incubation. **(B)** Rhodamine accumulation in promastigotes of sensitive (2001) and resistant (39, 41, R-5) isolates under ATP depletion after 15 minutes of incubation. **(C)** Calcein accumulation in promastigotes of sensitive (2001) and resistant (39, 41, R-5) isolates under normal condition after 60 minutes of incubation. **(D)** Calcein accumulation in promastigotes of sensitive (2001) and resistant (39, 41, R-5) isolates under ATP depletion after 30 minutes of incubation. **(E)** Calcein retention in promastigotes of sensitive (2001) and resistant (39, 41, R-5) isolates without probenecid. **(F)** Calcein retention in promastigotes of sensitive (2001) and resistant (39, 41, R-5) isolates with probenecid. Data shown are results from 1 experiment and representative of 3 independent experiments.

Using flow cytometry, we have assayed functional activity of ABC transporters *viz*. multidrug resistance protein (MRP) and multidrug resistance gene (MDR) in these *Leishmania* isolates. It is anticipated that cells showing a resistant phenotype would have higher level of energy dependent pump activity and therefore show less fluorescence as compared to cells with normal levels of energy dependent pumping activity (sensitive isolate). In the presence of pump modulators, both sensitive and resistant cells are expected to give similar level of fluorescence. We discriminated dead cells by addition of propidium iodide (5 μg/ml). We measured both cellular dye accumulation and dye retention under normal (promastigotes suspended and assayed in culture medium) as well as ATP depleted conditions. Time kinetic study of accumulation of R123 showed that a rapid uptake of R123 occurred in promastigotes of all the isolates, being maximal in the antimonial resistant isolate 39 (Figure 1A). With the addition of verapamil, a known MDR modulator, a consistent decrease was observed in all isolates except R-5 where two fold increase in R123 accumulation was observed. In order to assess whether ATP influenced this accumulation of R123, cellular ATP was depleted by the addition of NaN_3_ in the incubation buffer. The removal of ATP did not alter the R123 accumulation as net fluorescence in the presence or absence of ATP was no different (Figure1B). Once again, the addition of verapamil resulted in a decrease in R123 accumulation. This suggests that *Leishmania donovani* clinical isolates lack an energy-dependent classical MDR efflux system that can be reversed by verapamil. As can be seen from the accumulation assay, there is no hint of classical MDR pump activity, therefore, we did not perform the retention assay of rhodamine. It might be possible that the effect of verapamil on the accumulation of rhodamine in R5 may be related to the inhibition of a MDR-type transporter. With regard to the accumulation of calcein, 39 and 41 showed comparable levels of fluorescence, with 2001 having marginally higher level of fluorescence (Figure1C). On the other hand, R-5 had an almost 5 fold reduction in calcein accumulation as compared to other isolates. Addition of probenecid caused consistent increase in fluorescence in 2001, 39 and 41; maximal reversal was observed in 2001 and R-5 showed no alteration in accumulation of calcein. These results indicate that there is basal level of pumping activity in all clinical isolates tested but same concentration of probenecid cannot reverse pump activity to same level as seen in 39. This indicates that 39 may have some MRP pump activity. Under energy depleted conditions, calcein accumulation was greatly enhanced in 2001, 39, 41, the fold increase being 36.1, 44.0, and 27.3 respectively in comparison to their levels of accumulation in presence of ATP indicating that the pump is MRP like (Figure 1D). However R-5 showed only 2.6 fold increase in comparison to normal level accumulation. With addition of probenecid, the accumulation of calcein was marginally amplified in 2001 and 39, being 1.3 and 1.1 respectively indicating ATP depletion itself is sufficient to block the pump activity. However in 41, a 3.6 fold increase in fluorescence was observed, therefore, results suggest that in 41 there maybe change in ATPase level and it still used 10-15% leftover ATP for efflux after 30 minutes of NaN3 treatment. R-5 continued to show negligible fluorescence. One is tempted to attribute this to exceptionally higher level of pump activity. However, the non reversal with probenecid as also absent of any change following ATP depletion and disproves our hypothesis. It is conceivable that in R-5, the passive entry of calcein itself is restricted or esterase mediated conversion of calcein AM to calcein is altered. Assay of esterase activity in all isolates showed similar levels suggesting that lower calcein fluorescence observed in R-5 is not due to any esterase modification (data not shown). In the retention assay, cells were preloaded with calcein AM, washed and then incubated in calcein AM free medium in the presence or absence of probenecid. This allowed us to study the amount of calcein being retained within the parasites, which would indirectly reflect MRP activity. As seen in (Figure 1E and F) at 60 minutes, the sensitive isolate 2001, showed maximum retention of calcein as compared to the resistant isolates 39, 41, and R-5. Amongst the resistant isolates, 39 showed minimum calcein retention at each time point that was most pronounced at 60 minutes indicating maximum efflux activity that was validated by reversal with probenecid. In all isolates, probenecid enhanced retention of calcein indicating presence of MRP like pump.

### Thiol levels

Measurement of total intracellular thiol levels in energy depleted conditions in strains indicated that 39 and 41 show greater thiol level than 2001 and R-5 showed least level of thiol whereas under normal conditions thiol levels were 41 > 2001 > 39 > R-5 (Table ***3***)*.*

**Table 3:** **Times increase of total thiol levels in promastigotes of SAG resistant isolates 39, 41, R-5, with respect to the SAG sensitive isolate 2001 as calculated by the ratio of fluorescence of resistant isolates to sensitive isolate**

**Isolates**	**Normal condition**	**Energy depleted condition**
39	0.6	1.17
41	1.5	2.07
R-5	0.05	0.26

Data shown are results from 1 experiment and representative of 3 independent experiments.

### Amplification and overexpression of γ glutamylcysteine synthetase (GCS) gene

**Figure 2:**
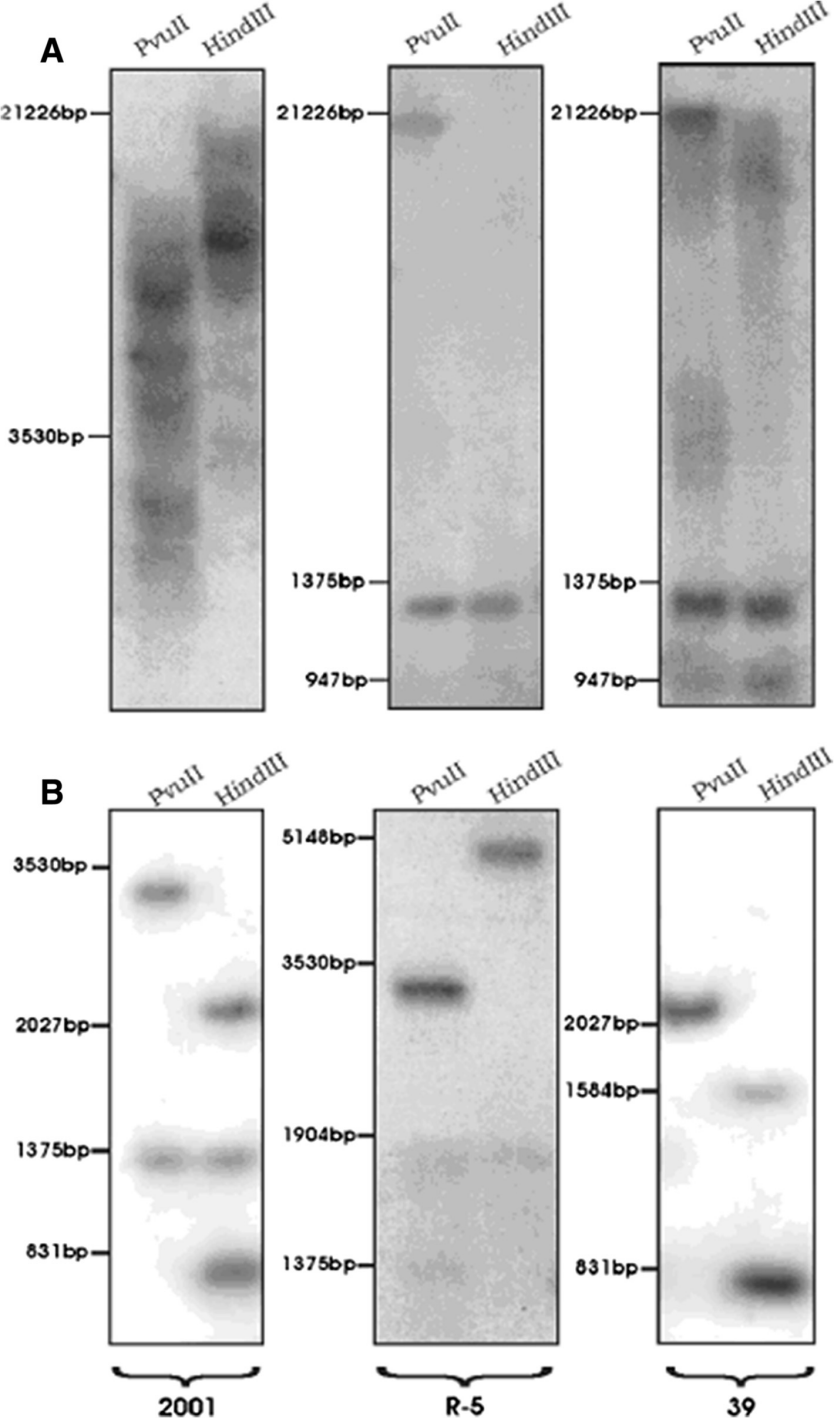
**Southern blot analysis for detection of (A) γ glutamylcysteine synthetase (** ***GCS*** **) gene amplification (B) same blot with α-tubulin gene probe.**

**Figure 3:**
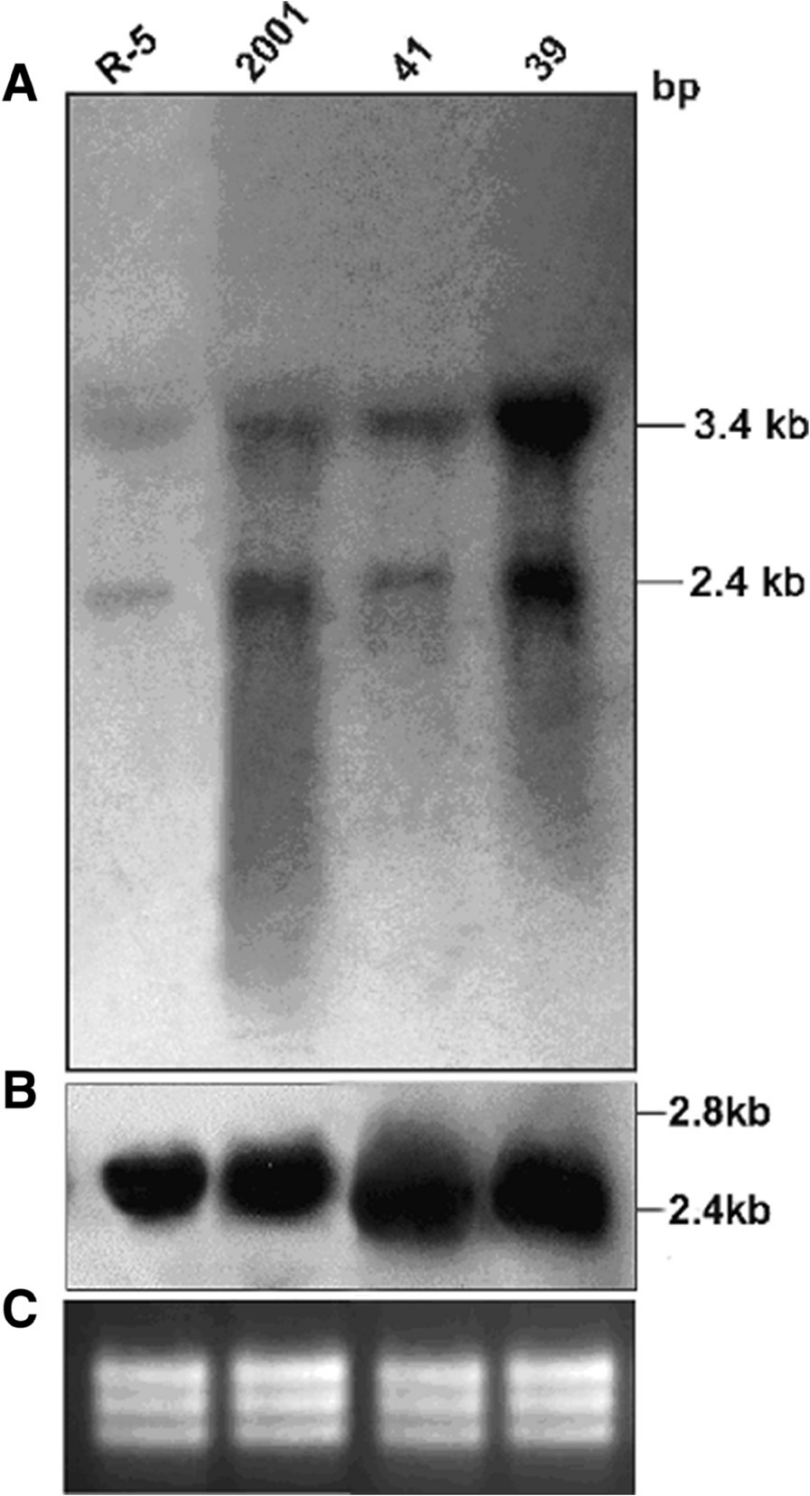
**Northern blot analysis for detection of (A) γ glutamylcysteine synthetase (** ***GCS*** **) gene overexpression (B) α-tubulin gene probe (C) EtBr stained rRNA bands showing equal loading.**

**Figure 4:**
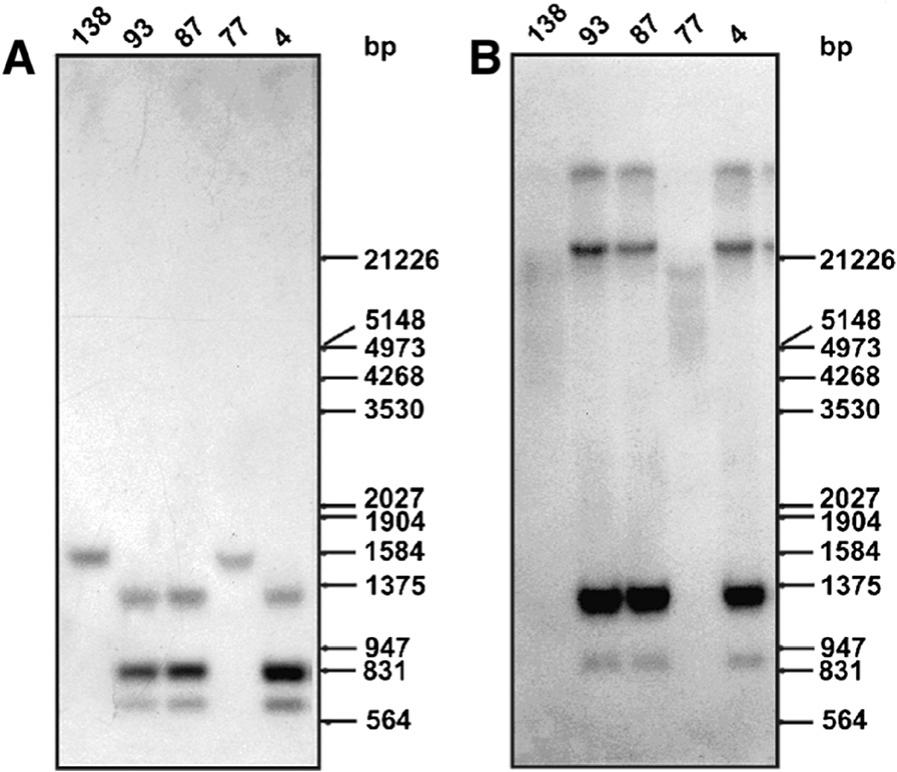
**Southern blot analysis for detection of (B) γ glutamylcysteine synthetase (** ***GCS*** **) gene amplification (A) same blot with α-tubulin gene probe.**

**Figure 5:**
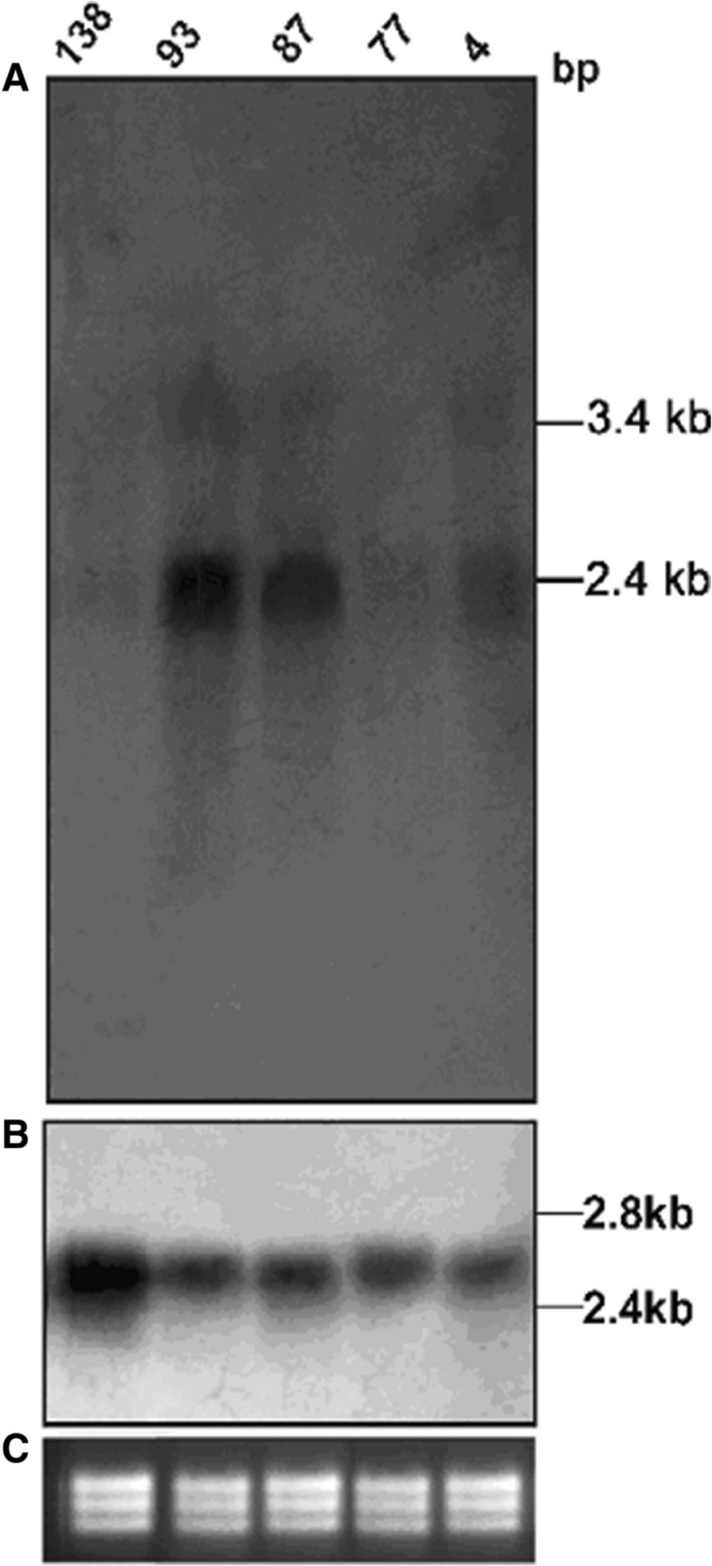
**Northern blot analysis for detection of (A) γ glutamylcysteine synthetase (** ***GCS*** **) gene overexpression (B) α-tubulin gene probe (C) EtBr stained rRNA bands showing equal loading.**

Equivalent amount of DNAs were digested with various restriction enzymes. Southern blotting using probe spanning γ glutamylcysteine synthetase (GCS) showed distinct amplification of the gene in SAG unresponsive isolates 39 and R-5 as opposed to responsive isolate 2001 (Figure 2A). Using the same probe, northern blotting showed an increased expression of two transcripts of 2.4 and 3.4 kb only in SAG unresponsive isolate 39 (Figure 3A). Amongst the isolates, 138, 93, 87, 77, 4; gene amplification was observed in 93, 87 and 4 (Figure 4B) and gene overexpression was seen in 93 and 87 (Figure 5A).

## Discussion

As illustrated in Table 1, we collected clinical isolates from the eastern region of India. We confirmed that no new strain responsible for drug unresponsiveness has emerged during this period [27]*.* This re- confirms the finding established during 1998-1999 [28]*.* We also established in these isolates that susceptibility to sodium antimony gluconate (SAG) as determined *in vitro* with intracellular amastigotes correlated with the clinical response [20]. The specific chemotherapeutic response of these isolates was persistent even after repeated passages in cultures as promastigotes and *in vivo* in experimental models [22] which again indicates that these isolates are truly refractory to SAG treatment in field conditions. Having established that resistance to SAG in clinical isolates is stable, our isolates are maintained in antimony free medium throughout. Although certain reports indicate that there is stage specificity to antimony susceptibility [29]*,* our study [20,22] established the fact that promastigotes are not altogether unresponsive to SAG but do show an intermediate susceptibility phenotype to antimony thus justifying the use of promastigotes as a preliminary screen to verify research methods and pinpoint potentially important genes using a stage that is easily grown in culture, and not contaminated by host material. Rhodamine/calcein uptake experiments are well established assays for detecting functional activity of MDR and MRP efflux pumps. These assays have extensively been used in cancer therapy where calcein AM, when used in a retention assay with MRP1 specific modulators, is able to reliably detect MRP functional activity [30] and similar strategy employed for *Leishmania* cells [31]*.* However, we must keep in mind that there is yet no published evidence that Sb and these substrates use the same transport pathways in *Leishmania.* In vinblastine and flavone resistant *Leishmania* strains the status of drug efflux pump MRP, has been studied using rhodamine 123 (Rh123) specific blockers to check the specificity of active efflux system, *viz* probenecid (blocker of MRP) and verapamil (blocker of MDR) [32,33]. Functional assays for analysis of MDR-related protein expression by flow cytometry have also been described [34]*.* The methodology for the determination of kinetics of these fluorochromes and modulators and parameters to use has been described and validated previously in the case of mammalian cells. We adapted this methodology to *Leishmania* promastigotes by performing experiments at 25°C and using higher cell number. A number of prior experiments by spectrofluorometer were done to reach the satisfactory concentration giving good accumulation/retention of fluorochrome within cells. To deplete intracellular ATP pools, the parasites were pretreated with 20 mM NaN3 and this is an established protocol used in *Leishmania* cells giving more than ~90% depletion [35]. Under chilled conditions, where endocytosis is prevented, as compared to sensitive, the resistant parasites showed decreased accumulation.

Using these well established functional assays, our data suggests that over functioning of MRP plays a role in generation of antimony resistance phenotype in some *L. donovani* clinical isolates. A striking difference from mammalian MRP2 localisation is that the MRPA transporter of *Leishmania* is an intracellular protein and it confers resistance to antimonials by sequestration of the metal thiol conjugates in an intracellular organelle located close to the flagellar pocket [36,37]. Resistance is due to enhanced efflux of the drug from the parasite from its flagellar pocket region, which is a major site for exocytosis-mediated process. We could establish that SAG unresponsive isolate 39, has developed resistance by an increased MRP like pump activity, and showed maximally increased levels of thiols as estimated by flow cytometry and detected by *GCS* gene overexpression. This indicates towards increased formation of metal thiol adducts and its sequetration by the ATP coupled MRP pump. It has been documented that antimony forms conjugate with cellular thiols and is then extruded by MRP [38]. In comparison to 39, in isolate 41, less MRP activity was observed which was greatly reversed by probenecid, therefore, results suggest that in 41 there maybe change in ATPase level and it still used 10-15% leftover ATP for efflux after 30 minutes of sodium azide treatment. SAG unresponsive isolate R-5, did not show any MRP pump activity and had lowest thiol levels and showed no overexpression of *GCS* gene in comparison to other SAG unresponsive isolates. This is in accordance to our analogy that membrane modification alone is the factor conferring resistance in this isolate [39]. Therefore, isolate 39 was confirmed to be maximally SAG resistant in comparison to isolate 41 and R-5.

Since, as established in arsenite resistant mutants, amplification of *GCS* gene is linked to an increase in the levels of GSH and TSH [40], the thiols cysteine, glutathione (GSH) and trypanothione (TSH) have been earlier measured in these same isolates by us by HPLC [41,42]. Quantification of thiols showed no differences in TSH levels between resistant and sensitive isolates, however, cysteine levels were increased in 39 when compared to 2001. GSH levels were increased in resistant isolates 39 and 41 when compared to 2001. Elevated levels of tryparedoxin peroxidase were also observed in these antimony unresponsive isolates (unpublished communication). Independent research groups in India who used our isolates also report that there is no classical MDR pump activity [43] and report amplification of MRPA, GCS and ODC both at genetic and transcriptional level. It has been shown that GCS expression is upregulated in SAG unresponsive parasites [44].

We added five new isolates namely 93, 77, 87, 4 and 138 to our repertoire (Table 2). At the time of their collection, because of the increasing antimonial drug resistance problem, treatment with SAG had been discontinued by our clinical collaborator. Isolates 87,4 and 138, were classified as being responsive solely due to the fact that these patients were from a region of India, namely Uttar Pradesh where so far antimonial drug resistance has not been reported to be present in comparison to the highly drug resistant endemic area of Bihar*.* So these isolates had actually not received SAG treatment by clinician but still have been categorized as responsive or unresponsive because of their origin from well acclaimed geographic region endemicity being resistant or sensitive. But when we checked for the *in vitro* drug sensitivity to SAG of these parasites in our laboratory (data not shown) we found that isolate 4 and 138 were indeed responsive/sensitive to drug thus conforming to the clinician’s criterion. However 87, turned out to be contrary to the clinician’s expectation. It showed *in vitro* drug resistance to SAG. It is possible, and we have frequently observed this in the field, that this patient 87 although hailing from a SAG sensitive area, would have taken this drug for treatment himself, as this drug is cheap and easily available over the counter. But since he was not cured, therefore showing *in vitro* drug resistance to SAG, reported to the clinician for subsequent treatment with amphotericin B. Isolates 93 and 77 were confirmed to be unresponsive to SAG in the laboratory.

SAG unresponsive isolate 93 showed both amplification and overexpression of GCS gene along with high intracellular thiol levels as assessed by Cell Tracker and MRP activity (data not shown). The other SAG unresponsive isolate 77 indicates towards resistance mechanism similar with R-5. Two SAG responsive isolates, 87 and 4 showed amplification of *GCS* gene with overexpression of gene only observed in isolate 87 which also showed increased total thiol levels. It has been reported in *Leishmania* that the amplification of gene does not always seem to lead to an increase in RNA levels. Thus one interesting observation brought out in our study is that the overexpression of GCS gene in Northern in isolate 87 resulted in resistance and also conforming to *in vitro* drug sensitivity profile of this isolate whereas the decreased expression of this gene as detected by Northern blotting in isolate 4, although showing corresponding gene amplification in Southern blotting, was followed by sensitivity to the drug.

## Conclusions

We speculate by analogy to the thiol linked proposed resistance mechanism in laboratory mutants, that antimony unresponsive clinical isolates are also associated with elevated thiols and subsequently with enhanced MRP activity.
